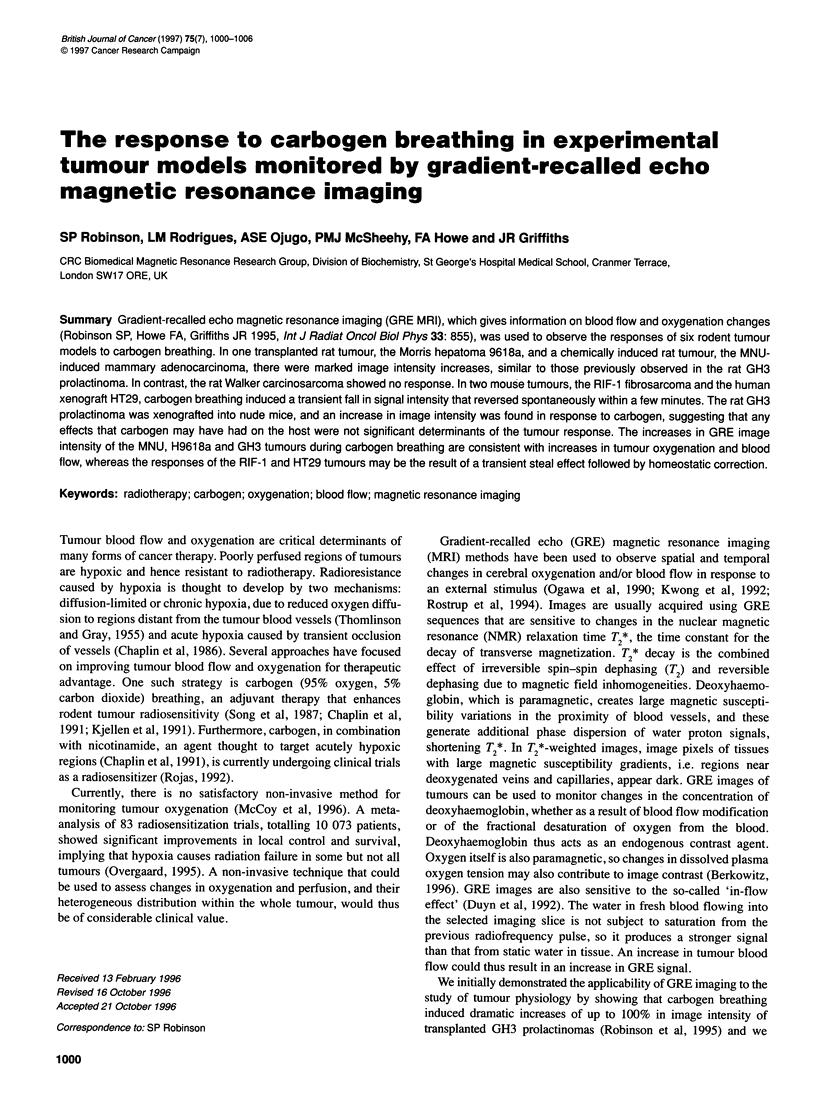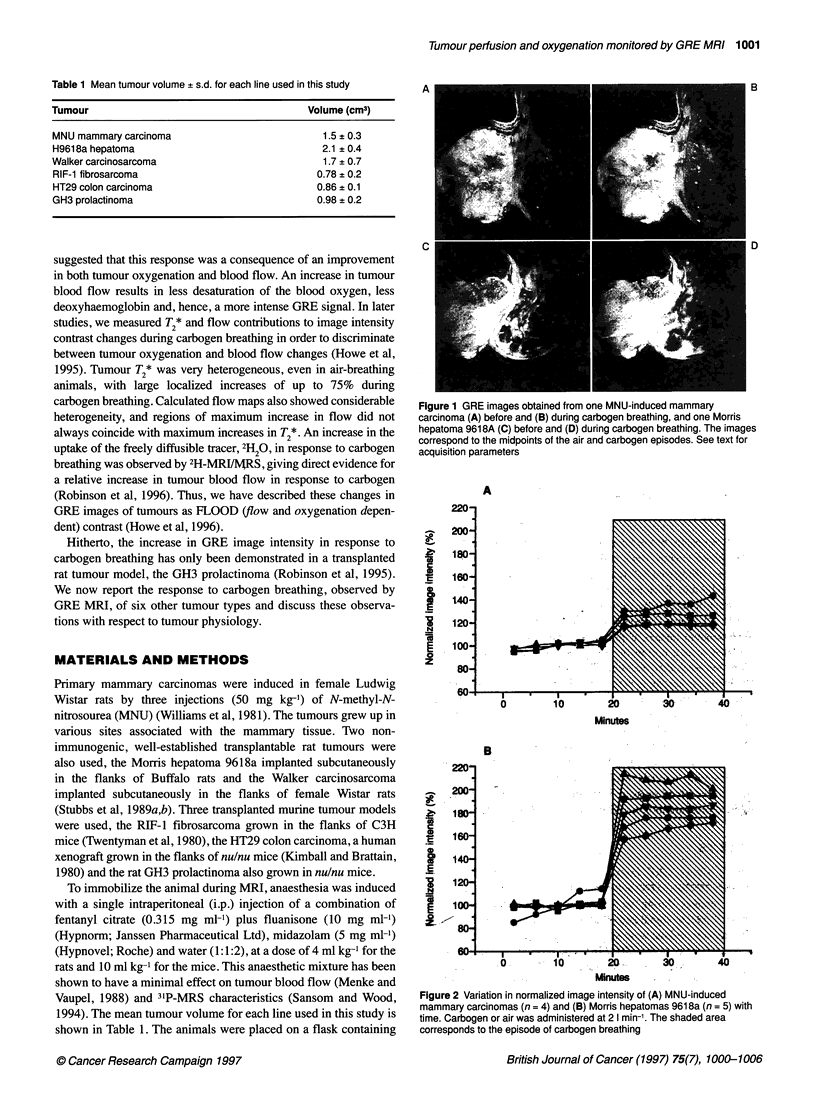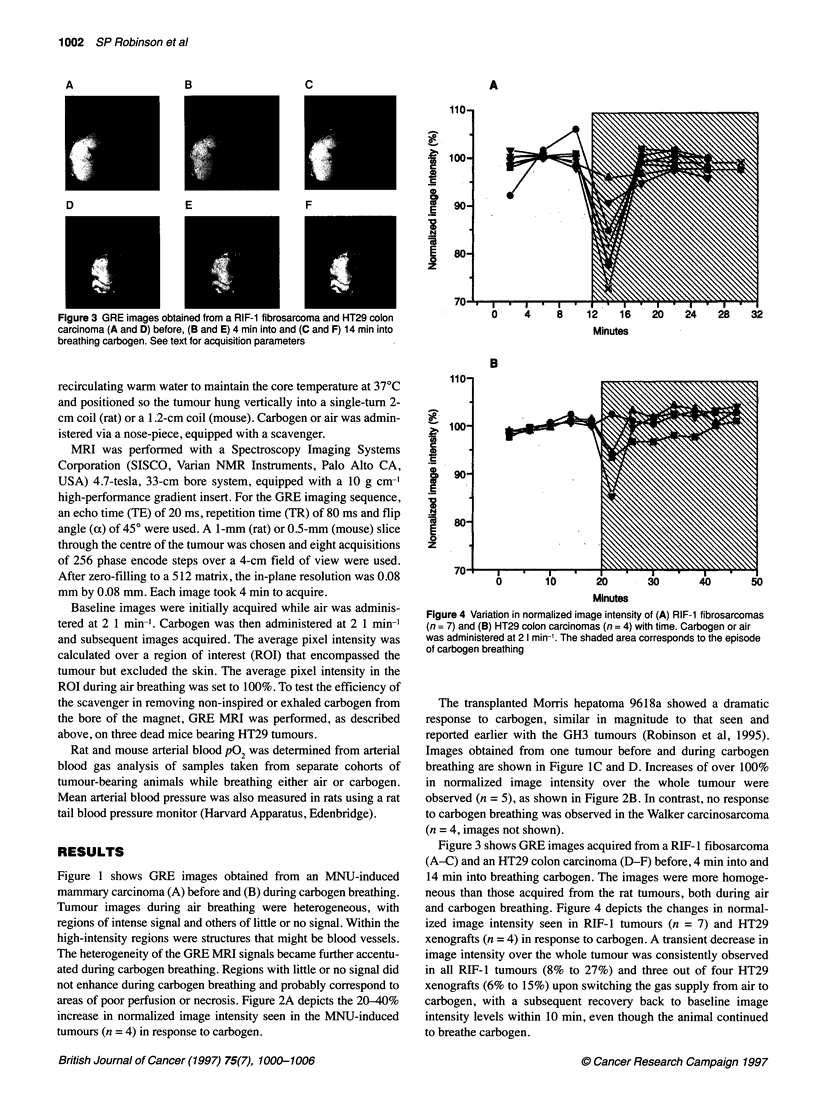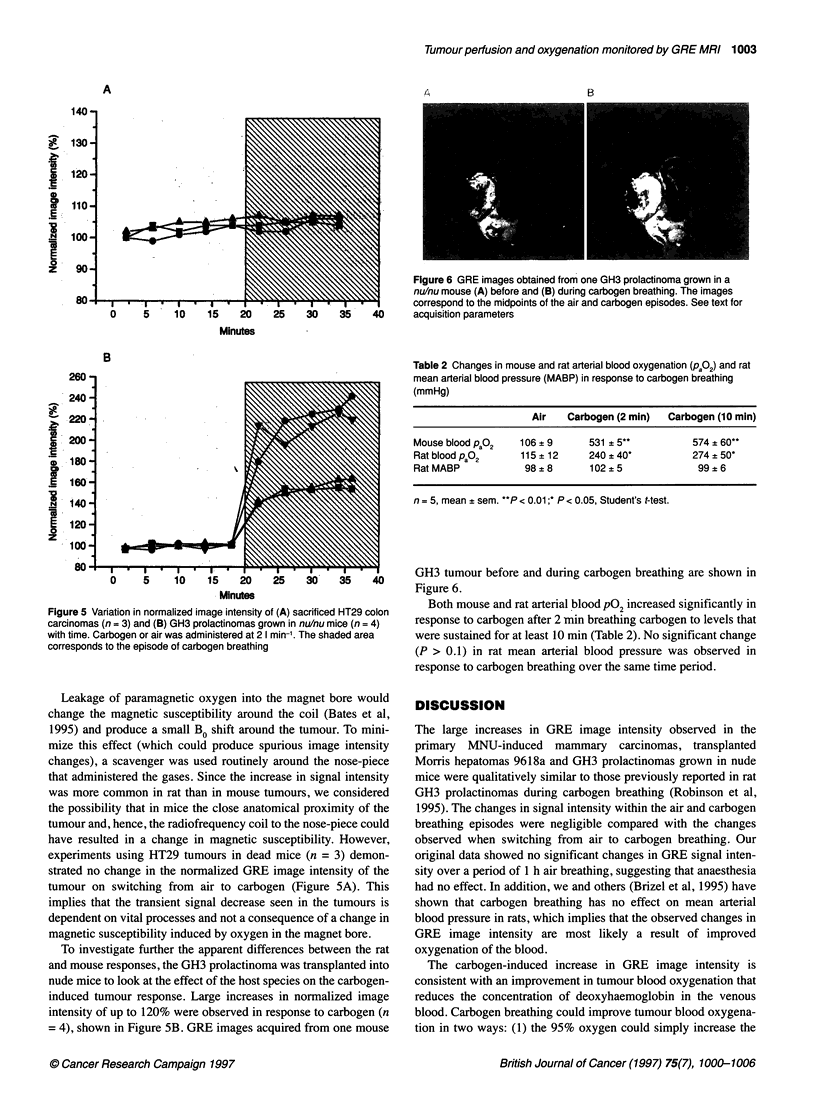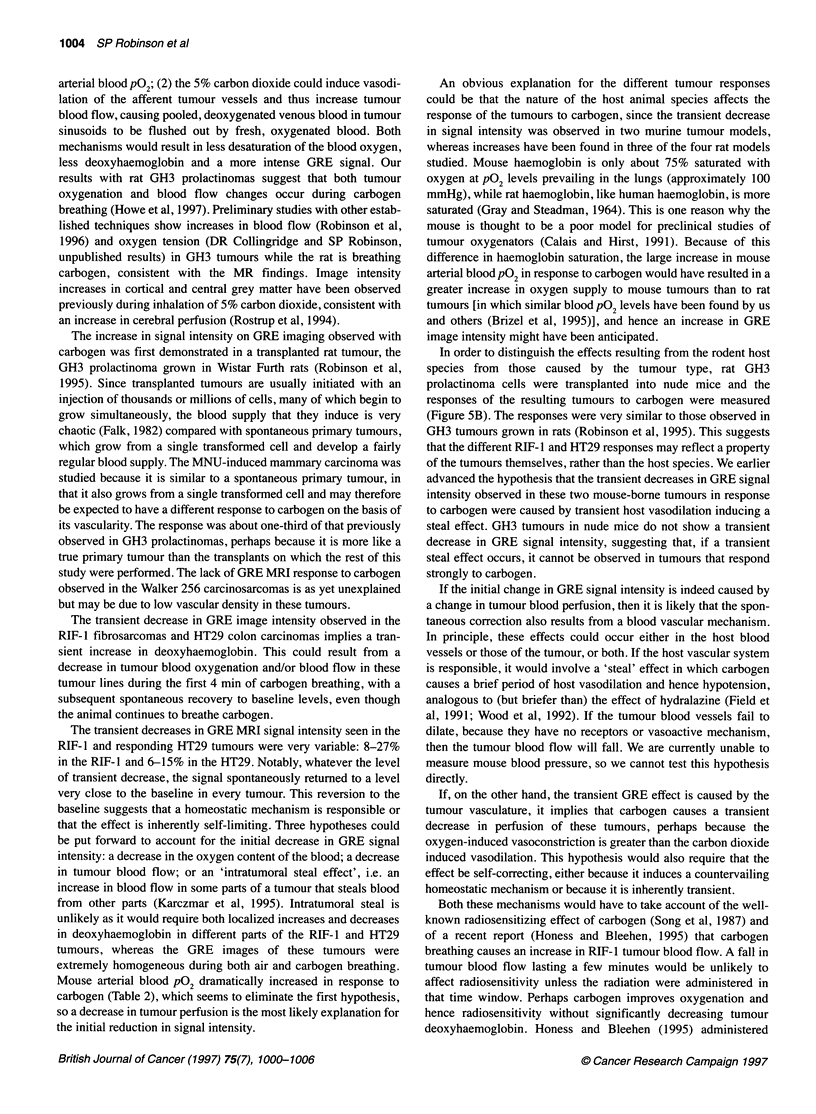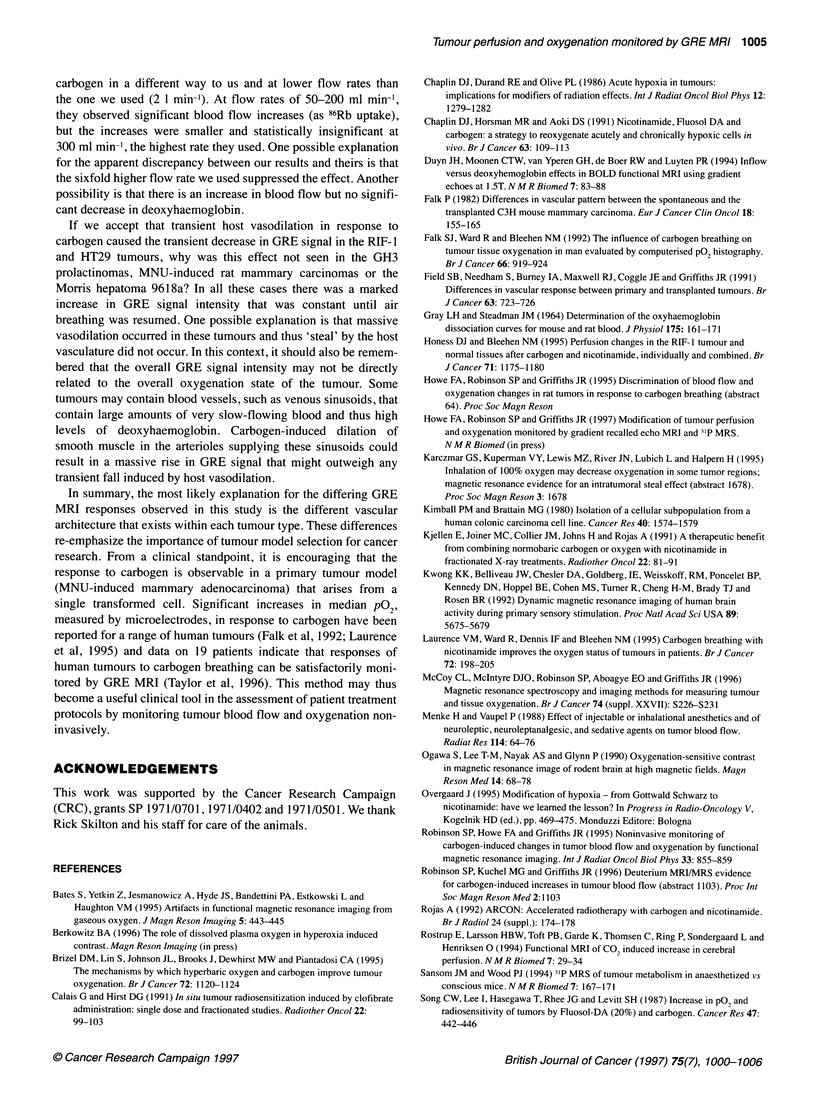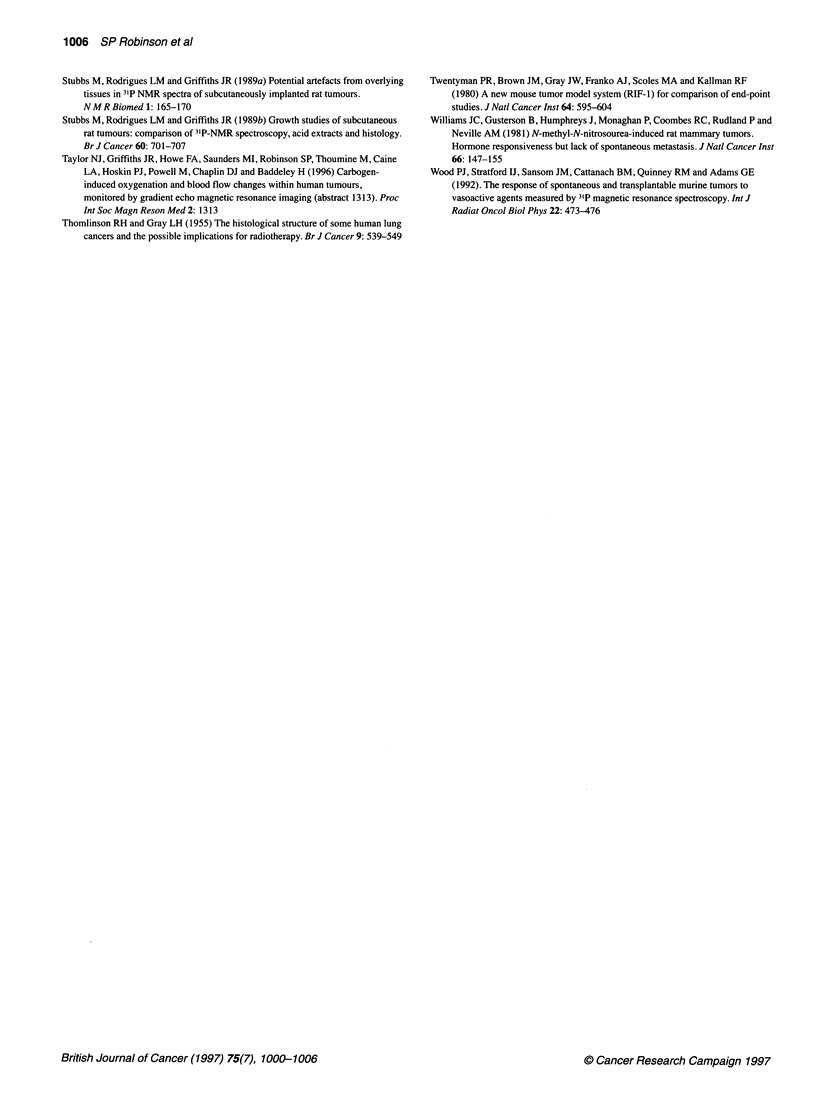# The response to carbogen breathing in experimental tumour models monitored by gradient-recalled echo magnetic resonance imaging.

**DOI:** 10.1038/bjc.1997.172

**Published:** 1997

**Authors:** S. P. Robinson, L. M. Rodrigues, A. S. Ojugo, P. M. McSheehy, F. A. Howe, J. R. Griffiths

**Affiliations:** Division of Biochemistry, St George's Hospital Medical School, London, UK.

## Abstract

**Images:**


*British Journal of Cancer* (1997) **75**(7), 1000–1006
© 1997 Cancer Research Campaign

### The response to carbogen breathing in experimental tumour models monitored by gradient-recalled echo magnetic resonance imaging

**SP Robinson, LM Rodrigues, ASE Ojugo, PMJ McSheehy, FA Howe and JR Griffiths**

CRC Biomedical Magnetic Resonance Research Group, Division of Biochemistry, St George's Hospital Medical School, Cranmer Terrace, London SW17 ORE, UK

**Summary** Gradient-recalled echo magnetic resonance imaging (GRE MRI), which gives information on blood flow and oxygenation changes (Robinson SP, Howe FA, Griffiths JR 1995, *Int J Radiat Oncol Biol Phys* **33**: 855), was used to observe the responses of six rodent tumour models to carbogen breathing. In one transplanted rat tumour, the Morris hepatoma 9618a, and a chemically induced rat tumour, the MNU-induced mammary adenocarcinoma, there were marked image intensity increases, similar to those previously observed in the rat GH3 prolactinoma. In contrast, the rat Walker carcinosarcoma showed no response. In two mouse tumours, the RIF-1 fibrosarcoma and the human xenograft HT29, carbogen breathing induced a transient fall in signal intensity that reversed spontaneously within a few minutes. The rat GH3 prolactinoma was xenografted into nude mice, and an increase in image intensity was found in response to carbogen, suggesting that any effects that carbogen may have had on the host were not significant determinants of the tumour response. The increases in GRE image intensity of the MNU, H9618a and GH3 tumours during carbogen breathing are consistent with increases in tumour oxygenation and blood flow, whereas the responses of the RIF-1 and HT29 tumours may be the result of a transient steal effect followed by homeostatic correction.

**Keywords:** radiotherapy; carbogen; oxygenation; blood flow; magnetic resonance imaging

Tumour blood flow and oxygenation are critical determinants of many forms of cancer therapy. Poorly perfused regions of tumours are hypoxic and hence resistant to radiotherapy. Radioresistance caused by hypoxia is thought to develop by two mechanisms: diffusion-limited or chronic hypoxia, due to reduced oxygen diffusion to regions distant from the tumour blood vessels (Thomlinson and Gray, 1955) and acute hypoxia caused by transient occlusion of vessels (Chaplin et al, 1986). Several approaches have focused on improving tumour blood flow and oxygenation for therapeutic advantage. One such strategy is carbogen (95% oxygen, 5% carbon dioxide) breathing, an adjuvant therapy that enhances rodent tumour radiosensitivity (Song et al, 1987; Chaplin et al, 1991; Kjellen et al, 1991). Furthermore, carbogen, in combination with nicotinamide, an agent thought to target acutely hypoxic regions (Chaplin et al, 1991), is currently undergoing clinical trials as a radiosensitizer (Rojas, 1992).

Currently, there is no satisfactory non-invasive method for monitoring tumour oxygenation (McCoy et al, 1996). A meta-analysis of 83 radiosensitization trials, totalling 10 073 patients, showed significant improvements in local control and survival, implying that hypoxia causes radiation failure in some but not all tumours (Overgaard, 1995). A non-invasive technique that could be used to assess changes in oxygenation and perfusion, and their heterogeneous distribution within the whole tumour, would thus be of considerable clinical value.

*Received 13 February 1996*
*Revised 16 October 1996*
*Accepted 21 October 1996*

*Correspondence to:* SP Robinson

Gradient-recalled echo (GRE) magnetic resonance imaging (MRI) methods have been used to observe spatial and temporal changes in cerebral oxygenation and/or blood flow in response to an external stimulus (Ogawa et al, 1990; Kwong et al, 1992; Rostrup et al, 1994). Images are usually acquired using GRE sequences that are sensitive to changes in the nuclear magnetic resonance (NMR) relaxation time $T_2$*, the time constant for the decay of transverse magnetization. $T_2$* decay is the combined effect of irreversible spin–spin dephasing ($T_2$) and reversible dephasing due to magnetic field inhomogeneities. Deoxyhaemoglobin, which is paramagnetic, creates large magnetic susceptibility variations in the proximity of blood vessels, and these generate additional phase dispersion of water proton signals, shortening $T_2$*. In $T_2$*-weighted images, image pixels of tissues with large magnetic susceptibility gradients, i.e. regions near deoxygenated veins and capillaries, appear dark. GRE images of tumours can be used to monitor changes in the concentration of deoxyhaemoglobin, whether as a result of blood flow modification or of the fractional desaturation of oxygen from the blood. Deoxyhaemoglobin thus acts as an endogenous contrast agent. Oxygen itself is also paramagnetic, so changes in dissolved plasma oxygen tension may also contribute to image contrast (Berkowitz, 1996). GRE images are also sensitive to the so-called 'in-flow effect' (Duyn et al, 1992). The water in fresh blood flowing into the selected imaging slice is not subject to saturation from the previous radiofrequency pulse, so it produces a stronger signal than that from static water in tissue. An increase in tumour blood flow could thus result in an increase in GRE signal.

We initially demonstrated the applicability of GRE imaging to the study of tumour physiology by showing that carbogen breathing induced dramatic increases of up to 100% in image intensity of transplanted GH3 prolactinomas (Robinson et al, 1995) and we

**Table 1** Mean tumour volume ± s.d. for each line used in this study

| Tumour | Volume ($cm^3$) |
|---|---|
| MNU mammary carcinoma | 1.5 ± 0.3 |
| H9618a hepatoma | 2.1 ± 0.4 |
| Walker carcinosarcoma | 1.7 ± 0.7 |
| RIF-1 fibrosarcoma | 0.78 ± 0.2 |
| HT29 colon carcinoma | 0.86 ± 0.1 |
| GH3 prolactinoma | 0.98 ± 0.2 |

suggested that this response was a consequence of an improvement in both tumour oxygenation and blood flow. An increase in tumour blood flow results in less desaturation of the blood oxygen, less deoxyhaemoglobin and, hence, a more intense GRE signal. In later studies, we measured $T_2$* and flow contributions to image intensity contrast changes during carbogen breathing in order to discriminate between tumour oxygenation and blood flow changes (Howe et al, 1995). Tumour $T_2$* was very heterogeneous, even in air-breathing animals, with large localized increases of up to 75% during carbogen breathing. Calculated flow maps also showed considerable heterogeneity, and regions of maximum increase in flow did not always coincide with maximum increases in $T_2$*. An increase in the uptake of the freely diffusible tracer, $^2H_2O$, in response to carbogen breathing was observed by $^2$H-MRI/MRS, giving direct evidence for a relative increase in tumour blood flow in response to carbogen (Robinson et al, 1996). Thus, we have described these changes in GRE images of tumours as FLOOD (*flow* and *o*xygenation *d*ependent) contrast (Howe et al, 1996).

Hitherto, the increase in GRE image intensity in response to carbogen breathing has only been demonstrated in a transplanted rat tumour model, the GH3 prolactinoma (Robinson et al, 1995). We now report the response to carbogen breathing, observed by GRE MRI, of six other tumour types and discuss these observations with respect to tumour physiology.

### MATERIALS AND METHODS

Primary mammary carcinomas were induced in female Ludwig Wistar rats by three injections (50 mg $kg^{-1}$) of *N*-methyl-*N*-nitrosourea (MNU) (Williams et al, 1981). The tumours grew up in various sites associated with the mammary tissue. Two non-immunogenic, well-established transplantable rat tumours were also used, the Morris hepatoma 9618a implanted subcutaneously in the flanks of Buffalo rats and the Walker carcinosarcoma implanted subcutaneously in the flanks of female Wistar rats (Stubbs et al, 1989*a*,*b*). Three transplanted murine tumour models were used, the RIF-1 fibrosarcoma grown in the flanks of C3H mice (Twentyman et al, 1980), the HT29 colon carcinoma, a human xenograft grown in the flanks of *nu/nu* mice (Kimball and Brattain, 1980) and the rat GH3 prolactinoma also grown in *nu/nu* mice.

To immobilize the animal during MRI, anaesthesia was induced with a single intraperitoneal (i.p.) injection of a combination of fentanyl citrate (0.315 mg $ml^{-1}$) plus fluanisone (10 mg $ml^{-1}$) (Hypnorm; Janssen Pharmaceutical Ltd), midazolam (5 mg $ml^{-1}$) (Hypnovel; Roche) and water (1:1:2), at a dose of 4 ml $kg^{-1}$ for the rats and 10 ml $kg^{-1}$ for the mice. This anaesthetic mixture has been shown to have a minimal effect on tumour blood flow (Menke and Vaupel, 1988) and $^{31}$P-MRS characteristics (Sansom and Wood, 1994). The mean tumour volume for each line used in this study is shown in Table 1. The animals were placed on a flask containing

**Figure 1** GRE images obtained from one MNU-induced mammary carcinoma (**A**) before and (**B**) during carbogen breathing, and one Morris hepatoma 9618A (**C**) before and (**D**) during carbogen breathing. The images correspond to the midpoints of the air and carbogen episodes. See text for acquisition parameters

**Figure 2** Variation in normalized image intensity of (**A**) MNU-induced mammary carcinomas (*n* = 4) and (**B**) Morris hepatomas 9618a (*n* = 5) with time. Carbogen or air was administered at 2 l $min^{-1}$. The shaded area corresponds to the episode of carbogen breathing

**Figure 3** GRE images obtained from a RIF-1 fibrosarcoma and HT29 colon carcinoma (**A** and **D**) before, (**B** and **E**) 4 min into and (**C** and **F**) 14 min into breathing carbogen. See text for acquisition parameters

**Figure 4** Variation in normalized image intensity of (**A**) RIF-1 fibrosarcomas ($n$ = 7) and (**B**) HT29 colon carcinomas ($n$ = 4) with time. Carbogen or air was administered at 2 l min$^{-1}$. The shaded area corresponds to the episode of carbogen breathing recirculating warm water to maintain the core temperature at 37°C and positioned so the tumour hung vertically into a single-turn 2-cm coil (rat) or a 1.2-cm coil (mouse). Carbogen or air was administered via a nose-piece, equipped with a scavenger.

MRI was performed with a Spectroscopy Imaging Systems Corporation (SISCO, Varian NMR Instruments, Palo Alto CA, USA) 4.7-tesla, 33-cm bore system, equipped with a 10 g cm$^{-1}$ high-performance gradient insert. For the GRE imaging sequence, an echo time (TE) of 20 ms, repetition time (TR) of 80 ms and flip angle ($\alpha$) of 45° were used. A 1-mm (rat) or 0.5-mm (mouse) slice through the centre of the tumour was chosen and eight acquisitions of 256 phase encode steps over a 4-cm field of view were used. After zero-filling to a 512 matrix, the in-plane resolution was 0.08 mm by 0.08 mm. Each image took 4 min to acquire.

Baseline images were initially acquired while air was administered at 2 l min$^{-1}$. Carbogen was then administered at 2 l min$^{-1}$ and subsequent images acquired. The average pixel intensity was calculated over a region of interest (ROI) that encompassed the tumour but excluded the skin. The average pixel intensity in the ROI during air breathing was set to 100%. To test the efficiency of the scavenger in removing non-inspired or exhaled carbogen from the bore of the magnet, GRE MRI was performed, as described above, on three dead mice bearing HT29 tumours.

Rat and mouse arterial blood $pO_2$ was determined from arterial blood gas analysis of samples taken from separate cohorts of tumour-bearing animals while breathing either air or carbogen. Mean arterial blood pressure was also measured in rats using a rat tail blood pressure monitor (Harvard Apparatus, Edenbridge).

### RESULTS

Figure 1 shows GRE images obtained from an MNU-induced mammary carcinoma (A) before and (B) during carbogen breathing. Tumour images during air breathing were heterogeneous, with regions of intense signal and others of little or no signal. Within the high-intensity regions were structures that might be blood vessels. The heterogeneity of the GRE MRI signals became further accentuated during carbogen breathing. Regions with little or no signal did not enhance during carbogen breathing and probably correspond to areas of poor perfusion or necrosis. Figure 2A depicts the 20–40% increase in normalized image intensity seen in the MNU-induced tumours ($n$ = 4) in response to carbogen.

The transplanted Morris hepatoma 9618a showed a dramatic response to carbogen, similar in magnitude to that seen and reported earlier with the GH3 tumours (Robinson et al, 1995). Images obtained from one tumour before and during carbogen breathing are shown in Figure 1C and D. Increases of over 100% in normalized image intensity over the whole tumour were observed ($n$ = 5), as shown in Figure 2B. In contrast, no response to carbogen breathing was observed in the Walker carcinosarcoma ($n$ = 4, images not shown).

Figure 3 shows GRE images acquired from a RIF-1 fibosarcoma (A–C) and an HT29 colon carcinoma (D–F) before, 4 min into and 14 min into breathing carbogen. The images were more homogeneous than those acquired from the rat tumours, both during air and carbogen breathing. Figure 4 depicts the changes in normalized image intensity seen in RIF-1 tumours ($n$ = 7) and HT29 xenografts ($n$ = 4) in response to carbogen. A transient decrease in image intensity over the whole tumour was consistently observed in all RIF-1 tumours (8% to 27%) and three out of four HT29 xenografts (6% to 15%) upon switching the gas supply from air to carbogen, with a subsequent recovery back to baseline image intensity levels within 10 min, even though the animal continued to breathe carbogen.

© Cancer Research Campaign 1997

**Figure 5** Variation in normalized image intensity of (**A**) sacrificed HT29 colon carcinomas ($n$ = 3) and (**B**) GH3 prolactinomas grown in *nu/nu* mice ($n$ = 4) with time. Carbogen or air was administered at 2 l min$^{-1}$. The shaded area corresponds to the episode of carbogen breathing

Leakage of paramagnetic oxygen into the magnet bore would change the magnetic susceptibility around the coil (Bates et al, 1995) and produce a small $B_0$ shift around the tumour. To minimize this effect (which could produce spurious image intensity changes), a scavenger was used routinely around the nose-piece that administered the gases. Since the increase in signal intensity was more common in rat than in mouse tumours, we considered the possibility that in mice the close anatomical proximity of the tumour and, hence, the radiofrequency coil to the nose-piece could have resulted in a change in magnetic susceptibility. However, experiments using HT29 tumours in dead mice ($n$ = 3) demonstrated no change in the normalized GRE image intensity of the tumour on switching from air to carbogen (Figure 5A). This implies that the transient signal decrease seen in the tumours is dependent on vital processes and not a consequence of a change in magnetic susceptibility induced by oxygen in the magnet bore.

To investigate further the apparent differences between the rat and mouse responses, the GH3 prolactinoma was transplanted into nude mice to look at the effect of the host species on the carbogen-induced tumour response. Large increases in normalized image intensity of up to 120% were observed in response to carbogen ($n$ = 4), shown in Figure 5B. GRE images acquired from one mouse GH3 tumour before and during carbogen breathing are shown in Figure 6.

**Figure 6** GRE images obtained from one GH3 prolactinoma grown in a *nu/nu* mouse (**A**) before and (**B**) during carbogen breathing. The images correspond to the midpoints of the air and carbogen episodes. See text for acquisition parameters

**Table 2** Changes in mouse and rat arterial blood oxygenation ($p_aO_2$) and rat mean arterial blood pressure (MABP) in response to carbogen breathing (mmHg)

| | Air | Carbogen (2 min) | Carbogen (10 min) |
|---|---|---|---|
| Mouse blood $p_aO_2$ | 106 ± 9 | 531 ± 5** | 574 ± 60** |
| Rat blood $p_aO_2$ | 115 ± 12 | 240 ± 40* | 274 ± 50* |
| Rat MABP | 98 ± 8 | 102 ± 5 | 99 ± 6 |

$n$ = 5, mean ± sem. **$P$ < 0.01;* $P$ < 0.05, Student's $t$-test.

Both mouse and rat arterial blood $pO_2$ increased significantly in response to carbogen after 2 min breathing carbogen to levels that were sustained for at least 10 min (Table 2). No significant change ($P$ > 0.1) in rat mean arterial blood pressure was observed in response to carbogen breathing over the same time period.

### DISCUSSION

The large increases in GRE image intensity observed in the primary MNU-induced mammary carcinomas, transplanted Morris hepatomas 9618a and GH3 prolactinomas grown in nude mice were qualitatively similar to those previously reported in rat GH3 prolactinomas during carbogen breathing (Robinson et al, 1995). The changes in signal intensity within the air and carbogen breathing episodes were negligible compared with the changes observed when switching from air to carbogen breathing. Our original data showed no significant changes in GRE signal intensity over a period of 1 h air breathing, suggesting that anaesthesia had no effect. In addition, we and others (Brizel et al, 1995) have shown that carbogen breathing has no effect on mean arterial blood pressure in rats, which implies that the observed changes in GRE image intensity are most likely a result of improved oxygenation of the blood.

The carbogen-induced increase in GRE image intensity is consistent with an improvement in tumour blood oxygenation that reduces the concentration of deoxyhaemoglobin in the venous blood. Carbogen breathing could improve tumour blood oxygenation in two ways: (1) the 95% oxygen could simply increase the

© Cancer Research Campaign 1997

arterial blood $pO_2$; (2) the 5% carbon dioxide could induce vasodilation of the afferent tumour vessels and thus increase tumour blood flow, causing pooled, deoxygenated venous blood in tumour sinusoids to be flushed out by fresh, oxygenated blood. Both mechanisms would result in less desaturation of the blood oxygen, less deoxyhaemoglobin and a more intense GRE signal. Our results with rat GH3 prolactinomas suggest that both tumour oxygenation and blood flow changes occur during carbogen breathing (Howe et al, 1997). Preliminary studies with other established techniques show increases in blood flow (Robinson et al, 1996) and oxygen tension (DR Collingridge and SP Robinson, unpublished results) in GH3 tumours while the rat is breathing carbogen, consistent with the MR findings. Image intensity increases in cortical and central grey matter have been observed previously during inhalation of 5% carbon dioxide, consistent with an increase in cerebral perfusion (Rostrup et al, 1994).

The increase in signal intensity on GRE imaging observed with carbogen was first demonstrated in a transplanted rat tumour, the GH3 prolactinoma grown in Wistar Furth rats (Robinson et al, 1995). Since transplanted tumours are usually initiated with an injection of thousands or millions of cells, many of which begin to grow simultaneously, the blood supply that they induce is very chaotic (Falk, 1982) compared with spontaneous primary tumours, which grow from a single transformed cell and develop a fairly regular blood supply. The MNU-induced mammary carcinoma was studied because it is similar to a spontaneous primary tumour, in that it also grows from a single transformed cell and may therefore be expected to have a different response to carbogen on the basis of its vascularity. The response was about one-third of that previously observed in GH3 prolactinomas, perhaps because it is more like a true primary tumour than the transplants on which the rest of this study were performed. The lack of GRE MRI response to carbogen observed in the Walker 256 carcinosarcomas is as yet unexplained but may be due to low vascular density in these tumours.

The transient decrease in GRE image intensity observed in the RIF-1 fibrosarcomas and HT29 colon carcinomas implies a transient increase in deoxyhaemoglobin. This could result from a decrease in tumour blood oxygenation and/or blood flow in these tumour lines during the first 4 min of carbogen breathing, with a subsequent spontaneous recovery to baseline levels, even though the animal continues to breathe carbogen.

The transient decreases in GRE MRI signal intensity seen in the RIF-1 and responding HT29 tumours were very variable: 8–27% in the RIF-1 and 6–15% in the HT29. Notably, whatever the level of transient decrease, the signal spontaneously returned to a level very close to the baseline in every tumour. This reversion to the baseline suggests that a homeostatic mechanism is responsible or that the effect is inherently self-limiting. Three hypotheses could be put forward to account for the initial decrease in GRE signal intensity: a decrease in the oxygen content of the blood; a decrease in tumour blood flow; or an 'intratumoral steal effect', i.e. an increase in blood flow in some parts of a tumour that steals blood from other parts (Karczmar et al, 1995). Intratumoral steal is unlikely as it would require both localized increases and decreases in deoxyhaemoglobin in different parts of the RIF-1 and HT29 tumours, whereas the GRE images of these tumours were extremely homogeneous during both air and carbogen breathing. Mouse arterial blood $pO_2$ dramatically increased in response to carbogen (Table 2), which seems to eliminate the first hypothesis, so a decrease in tumour perfusion is the most likely explanation for the initial reduction in signal intensity.

An obvious explanation for the different tumour responses could be that the nature of the host animal species affects the response of the tumours to carbogen, since the transient decrease in signal intensity was observed in two murine tumour models, whereas increases have been found in three of the four rat models studied. Mouse haemoglobin is only about 75% saturated with oxygen at $pO_2$ levels prevailing in the lungs (approximately 100 mmHg), while rat haemoglobin, like human haemoglobin, is more saturated (Gray and Steadman, 1964). This is one reason why the mouse is thought to be a poor model for preclinical studies of tumour oxygenators (Calais and Hirst, 1991). Because of this difference in haemoglobin saturation, the large increase in mouse arterial blood $pO_2$ in response to carbogen would have resulted in a greater increase in oxygen supply to mouse tumours than to rat tumours [in which similar blood $pO_2$ levels have been found by us and others (Brizel et al, 1995)], and hence an increase in GRE image intensity might have been anticipated.

In order to distinguish the effects resulting from the rodent host species from those caused by the tumour type, rat GH3 prolactinoma cells were transplanted into nude mice and the responses of the resulting tumours to carbogen were measured (Figure 5B). The responses were very similar to those observed in GH3 tumours grown in rats (Robinson et al, 1995). This suggests that the different RIF-1 and HT29 responses may reflect a property of the tumours themselves, rather than the host species. We earlier advanced the hypothesis that the transient decreases in GRE signal intensity observed in these two mouse-borne tumours in response to carbogen were caused by transient host vasodilation inducing a steal effect. GH3 tumours in nude mice do not show a transient decrease in GRE signal intensity, suggesting that, if a transient steal effect occurs, it cannot be observed in tumours that respond strongly to carbogen.

If the initial change in GRE signal intensity is indeed caused by a change in tumour blood perfusion, then it is likely that the spontaneous correction also results from a blood vascular mechanism. In principle, these effects could occur either in the host blood vessels or those of the tumour, or both. If the host vascular system is responsible, it would involve a 'steal' effect in which carbogen causes a brief period of host vasodilation and hence hypotension, analogous to (but briefer than) the effect of hydralazine (Field et al, 1991; Wood et al, 1992). If the tumour blood vessels fail to dilate, because they have no receptors or vasoactive mechanism, then the tumour blood flow will fall. We are currently unable to measure mouse blood pressure, so we cannot test this hypothesis directly.

If, on the other hand, the transient GRE effect is caused by the tumour vasculature, it implies that carbogen causes a transient decrease in perfusion of these tumours, perhaps because the oxygen-induced vasoconstriction is greater than the carbon dioxide induced vasodilation. This hypothesis would also require that the effect be self-correcting, either because it induces a countervailing homeostatic mechanism or because it is inherently transient.

Both these mechanisms would have to take account of the well-known radiosensitizing effect of carbogen (Song et al, 1987) and of a recent report (Honess and Bleehen, 1995) that carbogen breathing causes an increase in RIF-1 tumour blood flow. A fall in tumour blood flow lasting a few minutes would be unlikely to affect radiosensitivity unless the radiation were administered in that time window. Perhaps carbogen improves oxygenation and hence radiosensitivity without significantly decreasing tumour deoxyhaemoglobin. Honess and Bleehen (1995) administered

© Cancer Research Campaign 1997

carbogen in a different way to us and at lower flow rates than the one we used (2 l $min^{-1}$). At flow rates of 50–200 ml $min^{-1}$, they observed significant blood flow increases (as $^{86}Rb$ uptake), but the increases were smaller and statistically insignificant at 300 ml $min^{-1}$, the highest rate they used. One possible explanation for the apparent discrepancy between our results and theirs is that the sixfold higher flow rate we used suppressed the effect. Another possibility is that there is an increase in blood flow but no significant decrease in deoxyhaemoglobin.

If we accept that transient host vasodilation in response to carbogen caused the transient decrease in GRE signal in the RIF-1 and HT29 tumours, why was this effect not seen in the GH3 prolactinomas, MNU-induced rat mammary carcinomas or the Morris hepatoma 9618a? In all these cases there was a marked increase in GRE signal intensity that was constant until air breathing was resumed. One possible explanation is that massive vasodilation occurred in these tumours and thus 'steal' by the host vasculature did not occur. In this context, it should also be remembered that the overall GRE signal intensity may not be directly related to the overall oxygenation state of the tumour. Some tumours may contain blood vessels, such as venous sinusoids, that contain large amounts of very slow-flowing blood and thus high levels of deoxyhaemoglobin. Carbogen-induced dilation of smooth muscle in the arterioles supplying these sinusoids could result in a massive rise in GRE signal that might outweigh any transient fall induced by host vasodilation.

In summary, the most likely explanation for the differing GRE MRI responses observed in this study is the different vascular architecture that exists within each tumour type. These differences re-emphasize the importance of tumour model selection for cancer research. From a clinical standpoint, it is encouraging that the response to carbogen is observable in a primary tumour model (MNU-induced mammary adenocarcinoma) that arises from a single transformed cell. Significant increases in median $pO_2$, measured by microelectrodes, in response to carbogen have been reported for a range of human tumours (Falk et al, 1992; Laurence et al, 1995) and data on 19 patients indicate that responses of human tumours to carbogen breathing can be satisfactorily monitored by GRE MRI (Taylor et al, 1996). This method may thus become a useful clinical tool in the assessment of patient treatment protocols by monitoring tumour blood flow and oxygenation non-invasively.

### ACKNOWLEDGEMENTS

This work was supported by the Cancer Research Campaign (CRC), grants SP 1971/0701, 1971/0402 and 1971/0501. We thank Rick Skilton and his staff for care of the animals.

### REFERENCES

Bates S, Yetkin Z, Jesmanowicz A, Hyde JS, Bandettini PA, Estkowski L and Haughton VM (1995) Artifacts in functional magnetic resonance imaging from gaseous oxygen. *J Magn Reson Imaging* **5**: 443–445

Berkowitz BA (1996) The role of dissolved plasma oxygen in hyperoxia induced contrast. *Magn Reson Imaging* (in press)

Brizel DM, Lin S, Johnson JL, Brooks J, Dewhirst MW and Piantadosi CA (1995) The mechanisms by which hyperbaric oxygen and carbogen improve tumour oxygenation. *Br J Cancer* **72**: 1120–1124

Calais G and Hirst DG (1991) *In situ* tumour radiosensitization induced by clofibrate administration: single dose and fractionated studies. *Radiother Oncol* **22**: 99–103

Chaplin DJ, Durand RE and Olive PL (1986) Acute hypoxia in tumours: implications for modifiers of radiation effects. *Int J Radiat Oncol Biol Phys* **12**: 1279–1282

Chaplin DJ, Horsman MR and Aoki DS (1991) Nicotinamide, Fluosol DA and carbogen: a strategy to reoxygenate acutely and chronically hypoxic cells *in vivo*. *Br J Cancer* **63**: 109–113

Duyn JH, Moonen CTW, van Yperen GH, de Boer RW and Luyten PR (1994) Inflow versus deoxyhemoglobin effects in BOLD functional MRI using gradient echoes at 1.5T. *N M R Biomed* **7**: 83–88

Falk P (1982) Differences in vascular pattern between the spontaneous and the transplanted C3H mouse mammary carcinoma. *Eur J Cancer Clin Oncol* **18**: 155–165

Falk SJ, Ward R and Bleehen NM (1992) The influence of carbogen breathing on tumour tissue oxygenation in man evaluated by computerised $pO_2$ histography. *Br J Cancer* **66**: 919–924

Field SB, Needham S, Burney IA, Maxwell RJ, Coggle JE and Griffiths JR (1991) Differences in vascular response between primary and transplanted tumours. *Br J Cancer* **63**: 723–726

Gray LH and Steadman JM (1964) Determination of the oxyhaemoglobin dissociation curves for mouse and rat blood. *J Physiol* **175**: 161–171

Honess DJ and Bleehen NM (1995) Perfusion changes in the RIF-1 tumour and normal tissues after carbogen and nicotinamide, individually and combined. *Br J Cancer* **71**: 1175–1180

Howe FA, Robinson SP and Griffiths JR (1995) Discrimination of blood flow and oxygenation changes in rat tumors in response to carbogen breathing (abstract 64). *Proc Soc Magn Reson*

Howe FA, Robinson SP and Griffiths JR (1997) Modification of tumour perfusion and oxygenation monitored by gradient recalled echo MRI and $^{31}P$ MRS. *N M R Biomed* (in press)

Karczmar GS, Kuperman VY, Lewis MZ, River JN, Lubich L and Halpern H (1995) Inhalation of 100% oxygen may decrease oxygenation in some tumor regions; magnetic resonance evidence for an intratumoral steal effect (abstract 1678). *Proc Soc Magn Reson* **3**: 1678

Kimball PM and Brattain MG (1980) Isolation of a cellular subpopulation from a human colonic carcinoma cell line. *Cancer Res* **40**: 1574–1579

Kjellen E, Joiner MC, Collier JM, Johns H and Rojas A (1991) A therapeutic benefit from combining normobaric carbogen or oxygen with nicotinamide in fractionated X-ray treatments. *Radiother Oncol* **22**: 81–91

Kwong KK, Belliveau JW, Chesler DA, Goldberg, IE, Weisskoff, RM, Poncelet BP, Kennedy DN, Hoppel BE, Cohen MS, Turner R, Cheng H-M, Brady TJ and Rosen BR (1992) Dynamic magnetic resonance imaging of human brain activity during primary sensory stimulation. *Proc Natl Acad Sci* USA **89**: 5675–5679

Laurence VM, Ward R, Dennis IF and Bleehen NM (1995) Carbogen breathing with nicotinamide improves the oxygen status of tumours in patients. *Br J Cancer* **72**: 198–205

McCoy CL, McIntyre DJO, Robinson SP, Aboagye EO and Griffiths JR (1996) Magnetic resonance spectroscopy and imaging methods for measuring tumour and tissue oxygenation. *Br J Cancer* **74** (suppl. XXVII): S226–S231

Menke H and Vaupel P (1988) Effect of injectable or inhalational anesthetics and of neuroleptic, neuroleptanalgesic, and sedative agents on tumor blood flow. *Radiat Res* **114**: 64–76

Ogawa S, Lee T-M, Nayak AS and Glynn P (1990) Oxygenation-sensitive contrast in magnetic resonance image of rodent brain at high magnetic fields. *Magn Reson Med* **14**: 68–78

Overgaard J (1995) Modification of hypoxia – from Gottwald Schwarz to nicotinamide: have we learned the lesson? In *Progress in Radio-Oncology V*, Kogelnik HD (ed.), pp. 469–475. Monduzzi Editore: Bologna

Robinson SP, Howe FA and Griffiths JR (1995) Noninvasive monitoring of carbogen-induced changes in tumor blood flow and oxygenation by functional magnetic resonance imaging. *Int J Radiat Oncol Biol Phys* **33**: 855–859

Robinson SP, Kuchel MG and Griffiths JR (1996) Deuterium MRI/MRS evidence for carbogen-induced increases in tumour blood flow (abstract 1103). *Proc Int Soc Magn Reson Med* **2**:1103

Rojas A (1992) ARCON: Accelerated radiotherapy with carbogen and nicotinamide. *Br J Radiol* 24 (suppl.): 174–178

Rostrup E, Larsson HBW, Toft PB, Garde K, Thomsen C, Ring P, Sondergaard L and Henriksen O (1994) Functional MRI of $CO_2$ induced increase in cerebral perfusion. *N M R Biomed* **7**: 29–34

Sansom JM and Wood PJ (1994) $^{31}P$ MRS of tumour metabolism in anaesthetized *vs* conscious mice. *N M R Biomed* **7**: 167–171

Song CW, Lee I, Hasegawa T, Rhee JG and Levitt SH (1987) Increase in $pO_2$ and radiosensitivity of tumors by Fluosol-DA (20%) and carbogen. *Cancer Res* **47**: 442–446

© Cancer Research Campaign 1997

Stubbs M, Rodrigues LM and Griffiths JR (1989*a*) Potential artefacts from overlying tissues in $^{31}$P NMR spectra of subcutaneously implanted rat tumours. *N M R Biomed* **1**: 165–170

Stubbs M, Rodrigues LM and Griffiths JR (1989*b*) Growth studies of subcutaneous rat tumours: comparison of $^{31}$P-NMR spectroscopy, acid extracts and histology. *Br J Cancer* **60**: 701–707

Taylor NJ, Griffiths JR, Howe FA, Saunders MI, Robinson SP, Thoumine M, Caine LA, Hoskin PJ, Powell M, Chaplin DJ and Baddeley H (1996) Carbogen-induced oxygenation and blood flow changes within human tumours, monitored by gradient echo magnetic resonance imaging (abstract 1313). *Proc Int Soc Magn Reson Med* **2**: 1313

Thomlinson RH and Gray LH (1955) The histological structure of some human lung cancers and the possible implications for radiotherapy. *Br J Cancer* **9**: 539–549

Twentyman PR, Brown JM, Gray JW, Franko AJ, Scoles MA and Kallman RF (1980) A new mouse tumor model system (RIF-1) for comparison of end-point studies. *J Natl Cancer Inst* **64**: 595–604

Williams JC, Gusterson B, Humphreys J, Monaghan P, Coombes RC, Rudland P and Neville AM (1981) *N*-methyl-*N*-nitrosourea-induced rat mammary tumors. Hormone responsiveness but lack of spontaneous metastasis. *J Natl Cancer Inst* **66**: 147–155

Wood PJ, Stratford IJ, Sansom JM, Cattanach BM, Quinney RM and Adams GE (1992). The response of spontaneous and transplantable murine tumors to vasoactive agents measured by $^{31}$P magnetic resonance spectroscopy. *Int J Radiat Oncol Biol Phys* **22**: 473–476

*© Cancer Research Campaign 1997*